# The invisible malaria epidemic in Somalia: why molecular surveillance can no longer wait

**DOI:** 10.1186/s12936-026-06219-x

**Published:** 2026-11-10

**Authors:** Mohamed Mahdi Hashi, Abdisamad Abdihakim Aadam, Abdishakur Ahmed Mahad, Abdimalik Dahir Ali, Ilyas Abdullahi khalif

**Affiliations:** 1 Faculty of Health Sciences and Tropical Medicine , Somali National University , Mogadishu, Somalia; 2 Benaadir Research, Consultancy & Evaluation Center , Mogadishu, Somalia; 3 Center for Health Research and Innovation , Somali National University , Mogadishu, Somalia

**Keywords:** Malaria surveillance, Molecular epidemiology, Genomic surveillance, Antimalarial drug resistance, Somalia

## Abstract

Malaria remains a hidden public health challenge in Somalia, where weak surveillance systems and reliance on conventional diagnostics may underestimate transmission and disease complexity. The absence of molecular surveillance limits understanding of parasite diversity, drug resistance, and emerging transmission patterns. In the context of climate change, displacement, and fragile health systems, Somalia urgently needs genomic approaches integrated into malaria surveillance. Molecular surveillance can provide critical evidence to strengthen early detection, targeted interventions, and elimination strategies. Establishing these capacities now is essential to prevent an invisible malaria epidemic from becoming an uncontrolled public health crisis.

## Dear Editor,

Malaria control has entered a more precarious phase globally. In 2023, malaria cases exceeded those of 2022 by about 11 million, underscoring stalled progress despite renewed investments and expanding interventions [1]. In fragile settings such as Somalia, this stagnation is especially dangerous because routine surveillance systems are least able to detect changing transmission patterns, emerging resistance, and low-density infections that sustain onward spread. The result is not simply undercounting. It is an increasingly invisible epidemic hidden beneath weak diagnostics, conflict-related disruption, and delayed policy recognition. Evidence from neighboring Ethiopia and Eritrea underscores the importance of strengthening malaria surveillance in Somalia. Studies in Eritrea have documented the emergence and increasing prevalence of the *Plasmodium falciparum kelch13* (pfkelch*13*) R622I variant, which is associated with delayed parasite clearance and reduced susceptibility to artemisinin, alongside deletions of both *pfhrp2* and *pfhrp3*, which can compromise the sensitivity of histidine-rich protein 2 (HRP2)-based rapid diagnostic tests. Similar concerns have been reported in Ethiopia, where the R622I variant and parasites with *pfhrp2/pfhrp3* deletions have been identified. These regional findings highlight the potential for a dual challenge involving reduced antimalarial drug susceptibility and diagnostic evasion in the Horn of Africa. Although the extent to which these molecular markers circulate in Somalia requires direct investigation, the regional evidence supports prioritizing molecular surveillance of antimalarial resistance markers and *pfhrp2/pfhrp3* deletions to inform appropriate diagnostic and treatment policies [2, 3].

Somalia illustrates this problem with unusual clarity. Estimated malaria incidence in Somalia increased unevenly from 43.7 per 1,000 in 2010 to 52.2 per 1,000 in 2020, while broader East African analyses warn that underreporting is likely in conflict-affected settings such as Somalia because health-system weakness obscures the true burden [4, 5]. This means that the surveillance denominator itself is unstable: reported trends may reflect only the visible fraction of transmission. In such contexts, dependence on routine microscopy and rapid diagnostic tests alone is no longer sufficient, particularly when health access is poor and national analyses remain constrained by sparse subnational data [5].

The urgency is amplified by biological changes that conventional surveillance is poorly equipped to capture. Molecular methods have shown that many *Plasmodium falciparum* infections are submicroscopic yet still transmissible, and these infections predominate particularly in lower-transmission settings where elimination efforts are most vulnerable to hidden reservoirs [6]. At the same time, eastern Africa has become a focal region for artemisinin partial resistance, and major priorities now include expanded genomic surveillance for resistance mutations and more regular efficacy monitoring before treatment failure becomes clinically widespread [2, 3, 7]. Somalia cannot afford to wait for overt therapeutic collapse before acting. Molecular surveillance does not replace therapeutic efficacy studies, but it can detect early warning signals sooner, using dried blood spots and centralized laboratory analysis that are feasible even where field systems are constrained [8]. In such contexts, reliance on routine microscopy and rapid diagnostic tests (RDTs) alone may be insufficient, particularly given evidence from Ethiopia and Eritrea of *Plasmodium falciparum* parasites with deletions of both *pfhrp2* and *pfhrp3*, which can evade detection by HRP2-based RDTs. These regional findings underscore the urgency of establishing molecular surveillance in Somalia to determine whether such deletions are circulating locally, assess their potential impact on diagnostic accuracy, and guide appropriate malaria diagnostic and control strategies [2, 3]. Molecular surveillance in Somalia should prioritize *pfkelch13* mutations associated with artemisinin partial resistance, alongside *pfcrt* and *pfmdr1* polymorphisms associated with altered antimalarial drug susceptibility. Mutations in *pfdhfr* and *pfdhps* should also be investigated where sulfadoxine–pyrimethamine resistance is relevant. If dihydroartemisinin–piperaquine is included in Somalia’s current treatment policy, *plasmepsin 2/3* copy-number variation should also be considered as a marker associated with piperaquine resistance. These priorities should be informed by the antimalarial medicines currently recommended in Somalia and assessed alongside therapeutic efficacy studies. Updated, geographically representative molecular surveillance would help identify emerging resistance markers and support evidence-based treatment-policy decisions[9, 10].

Vector dynamics further strengthen the case for immediate action. In Somalia, *Anopheles stephensi* was detected in 2019, and its spread across the Horn of Africa threatens previous gains in malaria control, particularly in urban settings where routine entomological surveillance has been inconsistent and the timing of introduction is uncertain [4]. Without integrated molecular and entomological surveillance, Somalia risks responding to urban malaria resurgence after establishment rather than at introduction. That is a costly mistake in a country where conflict, displacement, and service disruption already impede epidemic intelligence. Across displacement settings, malaria surveillance is hindered by disrupted routine services, minimal data infrastructure, poor coordination, and cross-border complexity [11]. More broadly, conflict increases infectious-disease risk through population displacement, reduced surveillance, diagnostic delays, disrupted control programmes, and weakened health systems [12].

This is why Somalia deserves immediate scientific and policy attention. The country sits at the intersection of hidden parasitemia, emerging drug resistance, vector invasion, and fragility-driven under detection. Molecular surveillance offers a practical bridge between research and control: it can characterize parasite reservoirs, map resistance markers spatially, support rotating surveillance sites, and inform earlier policy adaptation before excess mortality accumulates [8]. In low-resource settings, the question is no longer whether molecular surveillance is sophisticated, but whether failing to deploy it is scientifically defensible.

Three priorities follow. First, Somalia’s malaria programme should integrate PCR- and sequencing-based surveillance into routine case detection through dried blood spot collection at a geographically distributed network of health facilities, with special emphasis on urban corridors, border zones, and displaced populations [8, 11]. Second, donors and multilateral partners should finance regional reference laboratory capacity, shared data repositories, and cross-border surveillance platforms linking Somalia with neighboring Horn of Africa states [8]. Third, molecular data must be coupled to actionable policy triggers, including targeted therapeutic efficacy studies, adaptive vector control, and rapid response to urban transmission signals associated with invasive vectors or displaced populations [4, 8].

Somalia’s malaria problem is not only one of burden, but of visibility. When surveillance misses low-density infection, emerging resistance, and shifting vector ecology, policy responds too late. Molecular surveillance can no longer be treated as optional research infrastructure. In Somalia, it is now a core requirement for credible malaria control.

## Data Availability

No datasets were generated or analyzed during the current study.
